# Impact of Infection Control Measures to Control an Outbreak of Multidrug-Resistant Tuberculosis in a Human Immunodeficiency Virus Ward, Peru

**DOI:** 10.4269/ajtmh.15-0712

**Published:** 2016-12-07

**Authors:** Eduardo Ticona, Luz Huaroto, Daniela E. Kirwan, Milagros Chumpitaz, César V. Munayco, Mónica Maguiña, Marco A. Tovar, Carlton A. Evans, Roderick Escombe, Robert H. Gilman

**Affiliations:** 1 Department of Infectious Diseases, Hospital Nacional Dos de Mayo, Lima, Peru; 2 Facultad de Medicina, Universidad Nacional Mayor de San Marcos, Lima, Peru; 3 Department of Microbiology, Hospital Nacional Dos De Mayo, Lima, Peru; 4 Department of Medical Microbiology, St George's Hospital, London, United Kingdom; 5 Department of Infectious Diseases and Immunity, Imperial College London, London, United Kingdom; 6 Preventive Medicine and Biometrics, Uniformed Services University of the Health Sciences, Bethesda, Maryland; 7 Asociacion Benéfica Proyectos en Informatica, Salud, Medicina, y Agricultura (PRISMA), Lima, Peru; 8 Innovation for Health and Development (IFHAD), Laboratory of Research and Development, Universidad Peruana Cayetano Heredia, Lima, Peru; 9 Wellcome Trust Centre for Global Health Research, Imperial College London, London, United Kingdom; 10 Laboratorio de Investigación en Enfermedades Infecciosas, Laboratorios de Investigación y Desarrollo, Facultad de Ciencias y Filosofía, Universidad Peruana Cayetano Heredia, Lima, Peru; 11 Department of International Health, Johns Hopkins Bloomberg School of Public Health, Baltimore, Maryland

## Abstract

Multidrug-resistant tuberculosis (MDRTB) rates in a human immunodeficiency virus (HIV) care facility increased by the year 2000—56% of TB cases, eight times the national MDRTB rate. We reported the effect of tuberculosis infection control measures that were introduced in 2001 and that consisted of 1) building a respiratory isolation ward with mechanical ventilation, 2) triage segregation of patients, 3) relocation of waiting room to outdoors, 4) rapid sputum smear microscopy, and 5) culture/drug–susceptibility testing with the microscopic-observation drug-susceptibility assay. Records pertaining to patients attending the study site between 1997 and 2004 were reviewed. Six hundred and fifty five HIV/TB–coinfected patients (mean age 33 years, 79% male) who attended the service during the study period were included. After the intervention, MDRTB rates declined to 20% of TB cases by the year 2004 (*P* = 0.01). Extremely limited access to antiretroviral therapy and specific MDRTB therapy did not change during this period, and concurrently, national MDRTB prevalence increased, implying that the infection control measures caused the fall in MDRTB rates. The infection control measures were estimated to have cost US$91,031 while preventing 97 MDRTB cases, potentially saving US$1,430,026. Thus, this intervention significantly reduced MDRTB within an HIV care facility in this resource-constrained setting and should be cost-effective.

## Introduction

The emergence of multidrug-resistant tuberculosis (MDRTB) and its interaction with human immunodeficiency virus (HIV) infection have complicated global efforts to control tuberculosis (TB).^1^,^2^ TB accelerates viral replication and progression to acquired immunodeficiency syndrome in HIV-infected patients, whereas HIV infection speeds progression to active TB after both long-standing latent and recently acquired infection.^3^–^5^

The superposition of the HIV and TB epidemics has led to high TB rates and TB outbreaks within HIV care facilities in cities including Madrid, Miami, and New York.^5^–^8^ In resource-constrained and wealthier countries alike, most of these outbreaks have involved MDRTB.^6^,^9^–^12^ In Latin America, high TB and MDRTB rates in association with HIV coinfection have been reported in Argentina^11^,^13^ and Peru. In Peru, a survey of patients receiving care at 10 large hospitals across Lima and Callao reported that 43% of patients with HIV/TB coinfection had MDRTB, compared with 3.9% of TB patients who were HIV negative.^14^

Strategies to combat nosocomial TB transmission have generally prioritized reducing the number of patient sources of infection, and infection control measures to limit the propagation of airborne TB within the hospital. These measures include a greater index of suspicion for TB, improving laboratory methods for TB diagnosis and drug-susceptibility testing, optimizing drug regimens to ensure effective treatment of individual cases, and isolation of infected patients.^9^,^15^,^16^ Employing such measures during nosocomial outbreaks has led to better management of outbreaks and has limited further transmission.^9^,^12^

In Peru, TB control measures during the 1990s centered on improving directly observed treatment, short-course (DOTS) for TB patients diagnosed by sputum smear microscopy, and in some settings, additionally the provision of isoniazid prophylaxis for HIV-positive patients without evidence of active TB. However, in some global settings, the impact of DOTS on TB and MDRTB control has been undermined by ineffective infection control programs, weak health systems infrastructure, and a lack of availability of second-line anti-TB drugs for HIV-positive patients.^17^

Analysis of *Mycobacterium tuberculosis* strains from patients with and without HIV coinfection at the Hospital Dos de Mayo (HDM) in Lima using restriction fragment length polymorphism identified “cluster” cases, suggesting transmission within this group of patients.^18^ Moreover, resistance rates were found to be increasing rapidly: in 1994–1995, 20% of evaluated patients had resistance to one or more drugs and 4% had MDRTB, rising to 71% resistance to one or more drugs including 41% MDR by 1997–1998.^19^–^21^ Despite evidence of ongoing nosocomial transmission of MDRTB in this hospital for several years,^22^ this was not incorporated in national TB reports for the year 2000.^23^,^24^ However, as a result of this evidence of nosocomial MDRTB transmission, the HDM administration authorized a team from the Infectious and Tropical Diseases department of HDM, with individual and institutional volunteers, to implement enhanced infection control measures. Herein, we describe these measures, their costs, and MDRTB rates before and after these measures were introduced.

## Methods

### Study design and ethics.

This study retrospectively assessed programmatically recorded data at the adult HIV care facility at the Dos De Mayo public tertiary referral hospital, Lima, Peru, from 1997 to 2004. Annually, this facility had an average of 310,948 outpatient visits and 360 hospitalizations of people at least 18 years of age, during this period. We examined records from all patients ≥ 18 years of age who were HIV positive and TB coinfected. Only patients with culture-positive samples were included in this study, to be able to classify patients according to culture-based drug susceptibility. HIV infection was defined by at least two positive HIV antibody test results: one in the hospital's onsite laboratory, and a confirmatory test performed in the Peruvian National Institutes of Health (NIH) reference laboratory. We aimed to compare these data before versus after infection control measures that were introduced to address a clinical need and to improve the standard of care, not for research purposes. The internationally accredited ethics committee at the nongovernmental organization Asociacion Benéfica Proyectos en Informatica, Salud, Medicina, y Agricultura (PRISMA), Lima, Peru, considered that as no human subject research was performed, retrospective analysis of these anonymous data did not require ethical approval.

### Data collection.

This study did not affect patients' routine clinical care that involved their nurses and/or doctors entering personal data into books. These data included sex, age, date(s) of outpatient attendance and/or admission to and discharge from the ward, date of HIV and/or TB diagnosis, and results of sputum smear microscopy, culture and susceptibility testing for first-line TB drugs. These data were digitized for the current research.

### Infection control measures.

The infection control measures were developed according to international recommendations^25^ and constituted 1) building a respiratory isolation ward with mechanical ventilation, 2) triage segregation of patients, 3) relocation of the outpatient waiting area from within to outside the building, 4) rapid sputum smear microscopy, and 5) culture with integrated drug-susceptibility testing with the Microscopic Observation Drug Susceptibility (MODS) assay. These components of the intervention are described below.

#### The respiratory isolation ward.

**Figure 1.**
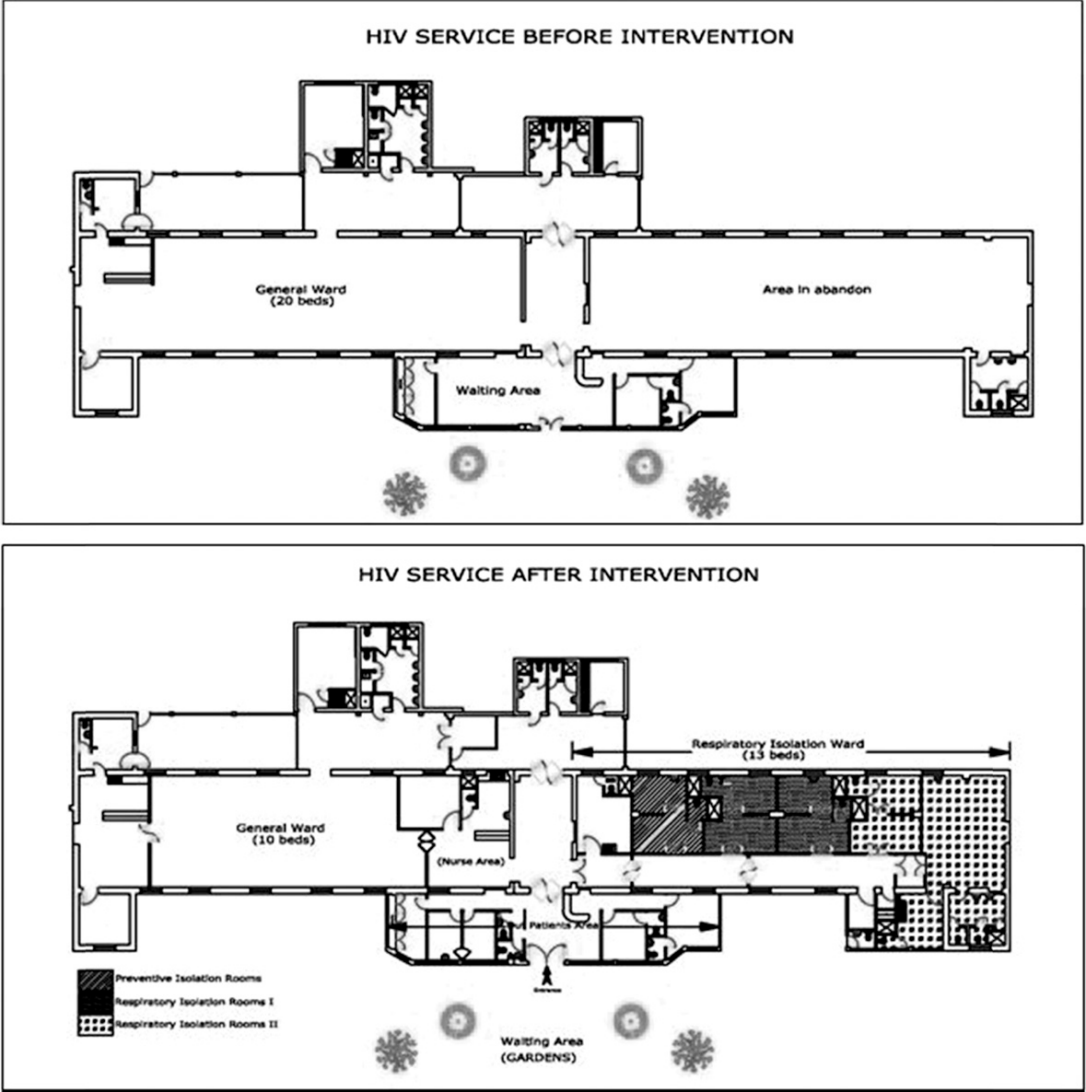
(**A**) Initial floorplan of the human immunodeficiency virus (HIV) service before the intervention. (**B**) Final layout of the respiratory isolation area at the HIV service, from 2002 to the present day.

The original HIV ward consisted of a single room measuring 35 × 8 × 4.5 m with 10 large windows that permitted a transverse flow of air providing high levels of natural ventilation.^26^ Enabled by donations, in 2000–2001, an adjacent unused building was converted into an isolation ward to which patients with suspected or proven TB were admitted instead of the original ward since 2001. The construction work transiently reduced the number of patients who could be admitted to the ward in 2001. The isolation ward initially consisted of three six-bedded rooms plus one single-patient room. After 4 months, the number of patients was reduced to four per room. In 2002, each four-bedded room was divided into a cluster of four individual rooms, each with a shared bathroom (Figure 1

). The third room continued to be shared by up to four patients. Patients with confirmed or suspected MDRTB were accommodated in one cluster of four individual rooms (Figure 1). The isolation ward incorporated a mechanical ventilation system, and all rooms had negative pressure and 6–8 changes/hour as recommended at that time.^27^,^28^ In addition, the waiting area for outpatients, although considered adequately ventilated, was relocated to the gardens adjacent to the outpatient clinic rooms, allowing greater distance between each patient and greater dilution of respiratory droplets.

#### Triage segregation of patients and waiting area relocation.

**Figure 2.**
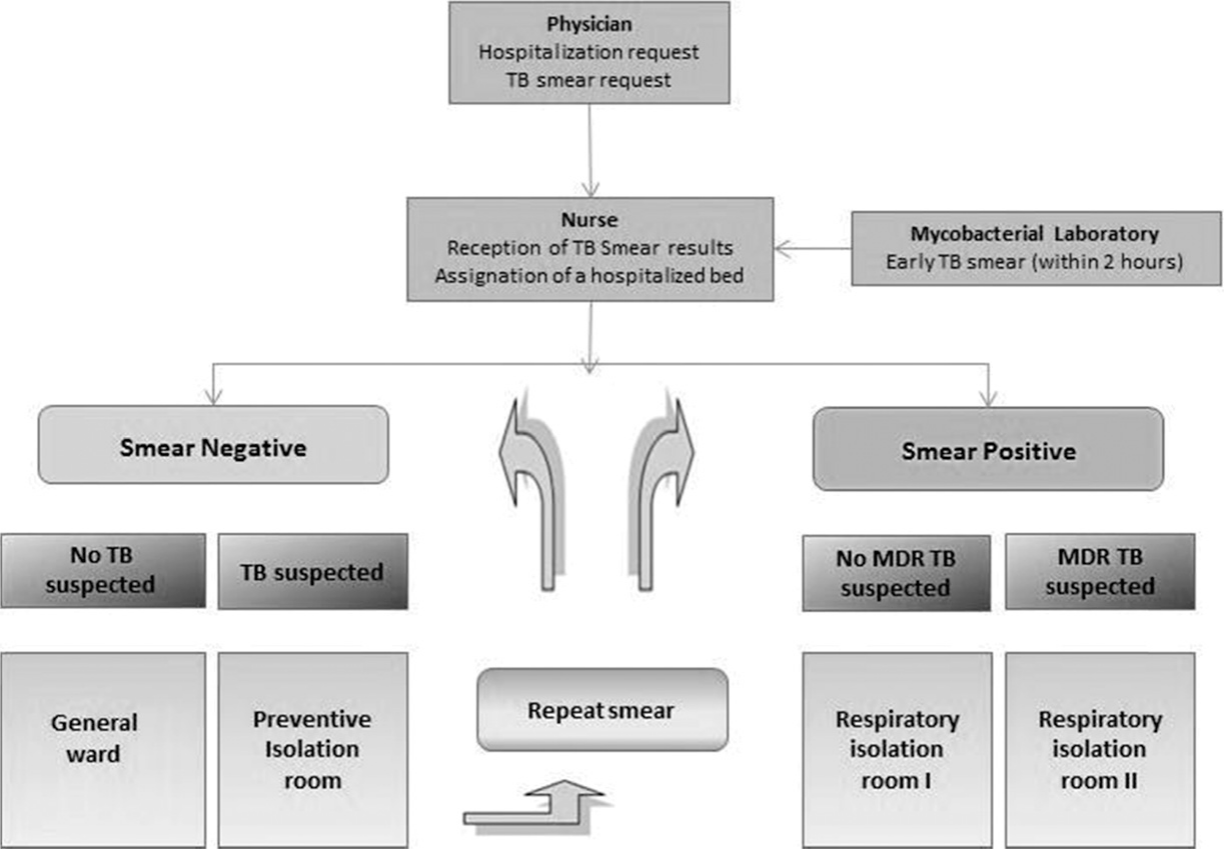
Triage flow diagram of admission procedures for the respiratory isolation area.

From 2001 on, all patients with negative sputum smear microscopy were hospitalized in the original open ward, and all patients with positive sputum smear microscopy were isolated in the respiratory isolation ward. However, it was noted that in some patients who had been smear negative on admission, there was ongoing clinical suspicion of TB and subsequent positive smear microscopy results. Consequently, in 2002, the admission protocol was modified (Figure 2

) so that all patients with cough suspected to be caused by TB were isolated in side rooms until TB was felt by the responsible clinician to have been confidently excluded on the basis of sequential sputum smear microscopy results, clinical assessment, and radiological evaluation. The MODS assay or epidemiological criteria were used to identify suspected MDRTB patients. These epidemiological criteria were failure to respond adequately to first-line treatment; contact with patients known to have MDRTB; early relapse after completion of first-line treatment; and multiple hospitalizations.

#### Rapid sputum smear microscopy and MODS.

In 2001, mandatory sputum smear microscopy at the time of outpatient review and/or hospitalization was established for all patients with respiratory symptoms. To facilitate this, rapid sputum smear microscopy was made available 12 hours/day, 6 days/week at the hospital laboratory. Forty percent of the patients had access to MODS assays, which permitted enhanced diagnosis in sputum smear-negative patients, and facilitated earlier isolation of patients with MDRTB.

Other practices that were unaffected by the infection control measures included natural ventilation by keeping windows open 24 hours a day and the obligatory use of highly protective N95 respirators by all health-care workers in contact with HIV-positive patients with suspected or proven pulmonary TB. These respirators have been available in Peru since 1997, and have been in widespread use since 1999.

### TB culture and drug-susceptibility testing.

Information about the availability of drug-susceptibility testing and first- and second-line drugs, for HDM patients and for all patients across Lima, are presented in Table 1. These data were obtained from the Ministry of Health, Lima, Peru (MINSA), and from records maintained at HDM.

TB culture was performed on solid Ogawa medium in the hospital laboratory as per standard practice. Positive isolates were sent to the Peruvian NIH laboratory for drug-susceptibility testing. Susceptibility testing for first-line TB drugs using the proportions method was available throughout the study period. In Lima, limited testing for susceptibility to second-line TB drugs became available from 1999 through the laboratory of the organization Partners in Health (PIH). These samples were sent to the Massachusetts State Laboratory Institute where testing was performed using the proportion method on 7H10 agar plates and, for pyrazinamide, the BACTEC method,^29^,^30^ and as a result, a significant turnaround time of several weeks could be expected. Moreover, testing was not available for patients attending sites (including HDM) that were geographically outside the PIH center's catchment area, which was restricted to north Lima. Susceptibility testing for second-line drugs became universally available through the Peruvian NIH laboratory in 2005, which was after the study period had ended.

For some patients attending HDM, MODS assay^31^,^32^ was performed at the Universidad Peruana Cayetano Heredia's laboratory. Its use during this study period was for research purposes only, as MODS was undergoing development and validation at that time, and the results were not recognized by the Peruvian NIH laboratory or Ministry of Health until 2005. As second-line drugs were approved and provided by the NIH, the results of the MODS assay were not used to inform treatment decisions. They were used to guide local infection control measures, however, as they were available to clinicians much more rapidly (usually 1–3 weeks) than those of the Peruvian NIH laboratory.

Molecular testing was not performed as part of this study. However, some of the participants included in this retrospective analysis gave informed written consent to provide sputum samples for a study of TB diagnostics and drug susceptibility that had prior ethical approval, the results of which are reported in an accompanying article in publication process.

### Data analysis.

For the purposes of analysis, a case of TB was defined as an individual who had a TB diagnosis confirmed by a positive *M. tuberculosis* culture and for whom phenotypic drug-susceptibility data were available. To assess the impact of TB infection control measures during fluctuations in ward occupancy and capacity, the percentage of patients with TB who had MDRTB before and after the intervention were compared using the unpaired Student's *t* test and the z-test of proportions.

These data were interpreted with reference to published national prevalence survey data defining the percentage of MDRTB among new and retreated patients.

### Cost analysis.

The number of MDRTB cases that the intervention prevented was estimated by calculating the proportion of MDRTB cases among total TB cases before, during, and after the intervention. The potential economic impact of the intervention was estimated by comparing potential savings from reducing the number of patients treated for MDRTB with the cost of the intervention. The cost of the intervention was calculated to include costs of new infrastructure and equipment plus annual maintenance. The potential cost of standardized second-line treatment of 18 months was calculated using costs previously reported,^29^,^33^ which include drugs, laboratory tests, and personnel.

## Results

A total of 912 patients with HIV/TB coinfection who had attended the HIV care facility during the study period were identified. Of these patients, 257 were excluded from analysis because of an absence of positive HIV test confirmation and/or because it was not possible to classify the patient as having drug-sensitive versus drug-resistant TB. Thus, 655 patients were included in the study. The mean age was 33 years, and 79% were male. No patients were taking sustained antiretroviral therapy (ART).

In 1997, 44% (52/117) HIV/TB–coinfected patients were diagnosed with MDRTB (Table 2). This percentage increased to peak at 56% (45/80 patients) in 2000, the year before the infection control measures took effect, and subsequently decreased to reach 20% (18/91 patients) in the year 2004.

**Figure 3.**
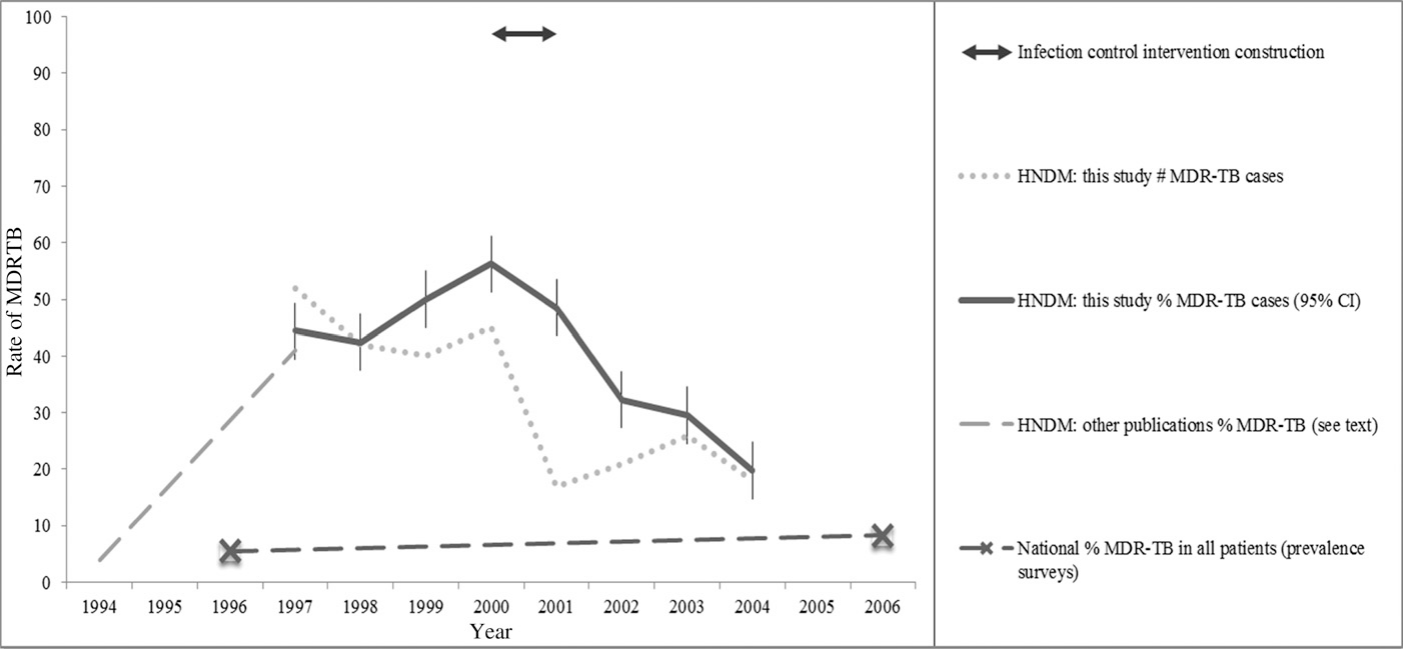
Trends in the rate of multidrug-resistant tuberculosis (MDRTB) among human immunodeficiency virus (HIV)/tuberculosis (TB)–coinfected patients before and after the implementation of the infection control intervention, HNDM = Hospital Nacional Dos de Mayo.

The trends in MDRTB rates among HIV/TB–coinfected patients are presented in Table 2 and illustrated in Figure 3

. For the 4 years before the infection control measures (1997–2000) combined, 48% of patients with TB had MDRTB (179/376), which was significantly higher (*P* < 0.0001) than for the 3 years after the infection control measures (2002–2004) when the MDRTB rate fell to 27% (65/244). Similarly, the number of MDRTB cases reduced significantly after the infection control measures were introduced (*P* < 0.0001).

During the study, national Peruvian prevalence surveys of randomly selected previously treated and untreated TB patients showed that in both of these groups, the MDRTB rates were increasing (Figure 3).^34^,^35^ Complete data were not recorded concerning MDRTB treatment at HDM during the period of this study. Two published studies, however, provide partial data. The first study recruited 30 patients with MDRTB from February 1999 to July 2000, 12 of whom had any access to second-line drugs.^36^ The second study recruited 50 patients at HDM with MDRTB between 2002 and 2003, only 15 of whom received any second-line drugs.^18^

The infection control measures prevented an estimated 97 cases of MDRTB in HIV-positive patients who received treatment from 2001 to 2004. The results of the economic analysis are given in Table 3. The infection control intervention was estimated to have caused an overall potential saving of US$1,430,026 if these cases had not been prevented and had instead been treated with second-line therapy.

## Discussion

This study shows that infection control measures designed to prevent TB transmission had a significant impact controlling an outbreak of MDRTB among HIV-positive patients in a tertiary referral hospital in Lima, Peru. In line with the lack of resources available in resource-constrained countries for such structural changes, these measures were developed with limited funding and introduced stepwise rather than simultaneously.

Table 1 indicates that the time period analyzed for this study was crucial in the fight against TB transmission at HDM and also across Lima as a whole. Very few patients with MDRTB were receiving appropriate treatment of their disease. At the onset of the study, both drug-susceptibility testing and provision of second-line drugs were scarce, only becoming universal right at the end of this time. Where they were available, the treatment regimens in use for MDRTB at that time were mostly standardized regimens, rather than individualized regimens that take into consideration an individual patient's drug-susceptibility data. Moreover, the availability of susceptibility testing and second-line treatment to HIV-positive patients, the subjects of this study, remained low throughout the study period. The appearance and rise in frequency of XDRTB is important and may reflect suboptimal management of TB patients. It is likely, therefore, that the changes in intervention control management implemented within the service made a significant contribution to the reduction in the proportion of MDRTB patients seen in our HIV/TB–coinfected population.

The striking and statisitically significant decrease in the proportion of MDRTB among HIV-positive patients with TB seen in this study is consistent with reports from Hospital Muñiz in Buenos Aires, Argentina, where the proportion of MDRTB cases among HIV-coinfected patients reduced from 16% in 1995 to 7% in 2001.^37^ In both hospitals, infection control measures included measures to improve detection of TB before and during admission, and isolation of patients with MDRTB.^6^ However, some methodological differences exist between the two studies. For example, in Hospital Muñiz, detection of drug resistance was performed using relatively rapid radiometric methods, and second-line TB medications were available for patients who required them. In contrast, our HIV service relied mainly on the proportions method for drug-susceptibility testing, so results were not available for several weeks or months. In addition, HIV-positive patients had severely limited access to second-line drugs as shown in Table 1 and described above. Furthermore, when second-line drugs were available, significant delays in obtaining and initiating these were almost universal, as reported previously.^29^ A small number of patients are known to have purchased second-line drugs privately^36^; however, these drug supplies were limited, were not prescribed according to recommended regimens, were often available only intermittently, and the duration for which they were taken is not known. This treatment was therefore inadequate. For these reasons, the occasional use of second-line drugs among our patients has not been included in this analysis.

Although nosocomial transmission of TB is believed to have been a long-standing problem in this HIV service, the outbreak of MDRTB documented by this report drew attention to this issue and prompted the introduction of rigorous infection control measures in the hospital as described. This infection control strategy reflected infection control policy recommendations from both the Centers for Disease Control and Prevention and World Health Organization, and therefore, our findings support those recommendations.^25^,^38^ In a study of 600 Peruvian TB patients over a 5-year period, 8.1% of patients with non-MDRTB were found to have acquired MDRTB during DOTS. This was 2.8 times more likely among HIV-positive patients (*P* = 0.001). In addition, acquired MDRTB was 34 times more likely to occur in clinics with higher proportions of patients with suspected MDRTB, suggesting transmission within these clinics.^39^

Among environmental control measures used in developing countries, natural ventilation is popular because it is considered to be effective and is less expensive than mechanical ventilation.^40^,^41^ The natural variation in airflow patterns during the day caused by local climate and other factors, the closing of windows at night, or the presence of highly infectious patients mean that its effectiveness can be highly variable, and complete elimination of infectious particles from the environment is not possible.^26^,^41^ However, isolation rooms equipped with negative pressure and mechanical ventilation typically guarantee an adequate and stable exchange of air, provided they are correctly designed and installed and well maintained. This ensures that contaminated air is channelled into a safe area alnd does not escape to other public places such as another ward, reception room, or doctor's office.^42^

This nosocomial outbreak of MDRTB occurred in an environment that was already equipped with natural ventilation facilitated by high ceilings and large windows. Additionally, infection control policies at the time advocated windows to be open 24 hours a day. However, in spite of the cross-ventilation, this policy was not followed due to various inevitable circumstances where patients needed the windows closed, such as cold weather, security, or sometimes when the privacy of the patients was compromised. It is clear that HIV-positive patients are highly susceptible to infection with TB,^43^–^45^ and our data further suggest that in this HIV ward for patients coinfected with TB, natural ventilation appeared to be insufficient to prevent transmission of TB. This evidence suggests that it may be necessary to use such additional measures in this type of setting.

The TB isolation ward was designed to provide six air changes per hour, consistent with recommendations at that time; however, since then, recommendations have increased to 12 changes/hour.^27^,^28^,^46^ This study demonstrates that in settings where achieving 12 air changes/hour is beyond resource availability, patient triage and segregation of infectious patients into rooms with lower levels of mechanical ventilation may considerably reduce MDRTB transmission.

Although the costs of the implementation and maintenance of the infection control measures described are significant, the reduction in the number of cases of MDRTB observed in this study produced a significant potential saving if second-line drugs had been available, estimated at US$1,430,026 during the study period. It is likely that the real savings are in fact much higher when the burden of MDRTB on quality of life^33^ and the economic costs to the family and society^47^ are considered. In addition, there are cost savings through a reduction in transmission of MDRTB in the community. This is especially important among the HIV-positive population given their greater susceptibility to TB infection as well as the higher rates of TB disease in HIV patients.

A limitation of this cost-effective model arises from the nature of TB infection in HIV-positive patients. The rapidly fatal nature of TB/HIV coinfection means that some patients may have succumbed to the disease before treatment with second-line drugs could be completed, or even initiated, thus reducing the expected expenditure on medications. This would depend upon the speed of detection of drug resistance and ease with which second-line drugs could be approved and obtained. Both of these factors were significant barriers to provision of timely care in Lima at this time as discussed above, and their effects are difficult to estimate. Moreover, this is likely to have been offset in part by the increased bacterial burden and aerosolization generated by a small number of individual “super spreaders” who are responsible for a significant proportion of transmission in hospital settings.^48^

Particularly in developing countries, the combination of natural and mechanical ventilation in care facilities, provision of rapid sputum smear microscopy or other rapid diagnostic tests such as MODS or the Xpert MTB/RIF assay (Cepheid, USA), and patient isolation appears to be a useful and cost-effective strategy to limit nosocomial transmission of TB within HIV wards. It is not possible to quantify the relative contribution of the separate components of the intervention on the reduction in transmission, as the intervention was implemented as an integrated package. In settings such as this where both TB and HIV are endemic and patient numbers are large, it may not be possible to isolate all patients with HIV, and thus the use of an algorithm to facilitate isolation of patients coinfected with TB or suspected TB (Figure 2) would represent a rational use of resources. This policy could be particularly useful in settings where MDRTB and/or extensively drug-resistant TB (XDRTB) are common.

The retrospective nature of this study and ethical considerations preclude the use of a concurrent control group to enable more rigorous evaluation of the infection control measures. Although the possibility that the temporal changes observed may have been due to other factors affecting TB transmission during the study period cannot be excluded, the marked reduction in numbers of patients diagnosed with MDRTB after the intervention suggests that the change was likely to be due to the infection control measures. Moreover, the patients admitted to the ward did not receive ART and very few received second-line drugs for TB, and the national MDRTB rates changed little during the study; implying these factors cannot therefore explain the rapid reduction in MDRTB rates that were seen.

A separate study was conducted from 2002 to 2004 evaluating upper-room ultraviolet light (UV) to prevent the transmission of TB.^49^ During this period, the isolation rooms had upper-room UV germicidal lights as part of the study. This may also have contributed to our findings; again, it is not possible to quantify the magnitude of its effect, particularly given that it was not consistently used during the intervention period.

**Figure 4.**
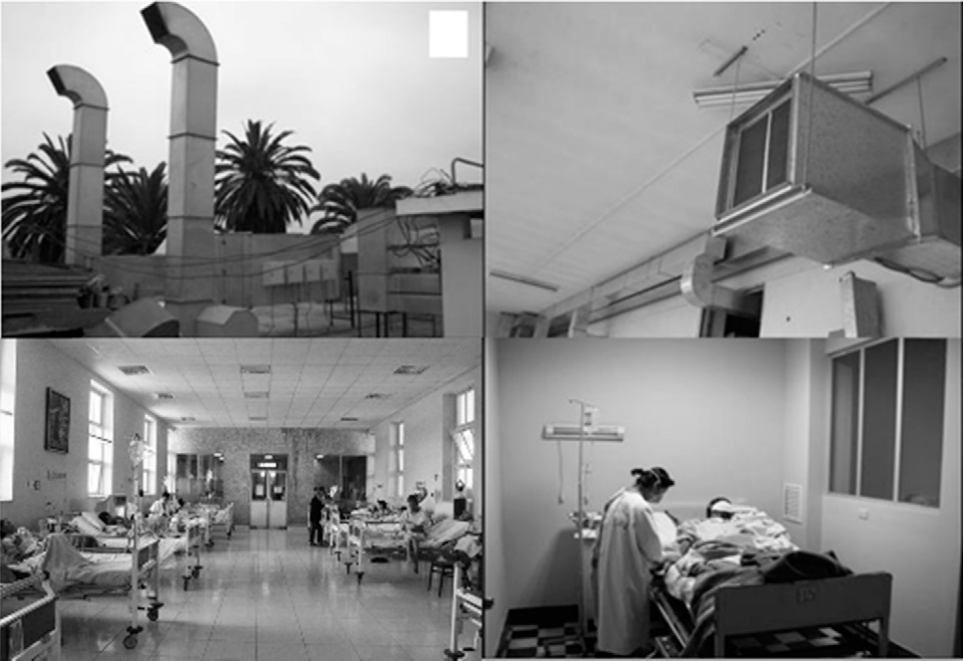
Photographs of air-elimination ducts (upper left); air-injection motor and filter (upper right); main ward with natural ventilation (lower left); respiratory isolation room (lower right).

In conclusion, this study has demonstrated that the implementation of a comprehensive TB control strategy including a natural and mechanical ventilation and other measures was associated with a marked reduction in nosocomial transmission of MDRTB in an HIV ward (Figure 4

). We also showed that these infection control measures were sustainable for several years in this resource-constrained setting. This is contrary to commonly held beliefs that respiratory isolation is almost impossible in such settings due to cost limitations, whereas in fact, the cost-effectiveness analysis indicates that this strategy has the potential to lead to considerable savings that may justify the initial economic investment.

## Figures and Tables

**Table 1:**
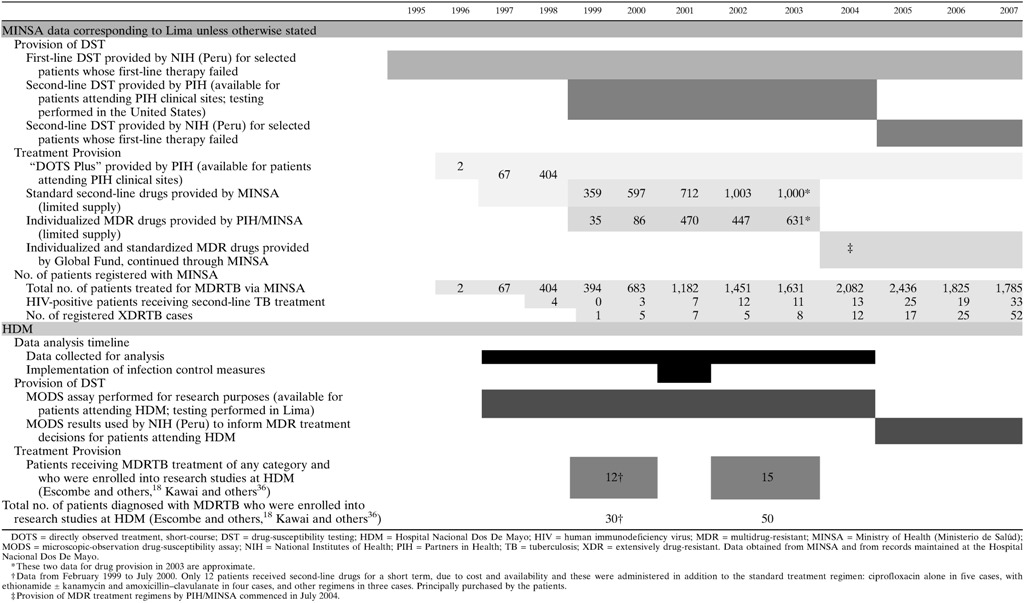
Provision of DST to second-line TB drugs and of second-line TB treatment in Lima, Peru, 1995–2007

**Table 2:** Annual numbers of patients with HIV/TB coinfection seen at the HIV service, and rates of MDRTB among these patients

		Year							
		Preintervention				Construction	Intervention		
		1997	1998	1999	2000	2001	2002	2003	2004
Patients with HIV/TB coinfection (all cases, drug-susceptible and MDRTB)	117	99	80	80	35	65	88	91
Patients with HIV/TB coinfection, non-MDRTB	65	57	40	35	18	44	62	73
Patients with HIV/MDRTB coinfection	52	42	40	45	17	21	26	18
% of total HIV/TB coinfection	44	42	50	56	49	32	30	20
Average (%) of total HIV/TB coinfection	48				49	27		
Ratio, MDR:non-MDR case	0.80	0.74	1.00	1.29	0.94	0.48	0.42	0.25
Average ratio, MDR:non-MDR case	0.91				0.94	0.36		
Predicted no. of MDRTB cases if the intervention had not occurred and the baseline MDRTB rate had continued	NA	NA	NA	NA	NA	40	56	66
Estimated no. of MDRTB cases prevented by the intervention	NA	NA	NA	NA	NA	19	30	48

HIV = human immunodeficiency virus; MDR = multi-drug resistant; NA = not applicable; TB = tuberculosis.

**Table 3:** Cost-effectiveness analysis of the implementation of the respiratory isolation ward

	Cost per unit (USD)	Unit	Total cost (USD)
57 MDRTB treatment^29^,^33^	15,681	97*	1,521,057
Implementation of environmental control measures			
First building	57,887	1	57,887
Final building after 1 year	25,000	1	25,000
Maintenance cost	2,036	4†	8,144
Total cost			91,031
Overall savings			1,430,026

MDRTB = multi-drug resistant tuberculosis; USD = U.S. dollar.

* Number of cases averted during the study period.

† Duration of the study period in years.
